# South Pole–Aitken massive impact 4.25 billion years ago revealed by Chang'e-6 samples

**DOI:** 10.1093/nsr/nwaf103

**Published:** 2025-03-20

**Authors:** Bin Su, Yi Chen, Zeling Wang, Di Zhang, Haojie Chen, Sheng Gou, Zongyu Yue, Yanhong Liu, Jiangyan Yuan, Guoqiang Tang, Shun Guo, Qiuli Li, Yang-Ting Lin, Xian-Hua Li, Fu-Yuan Wu

**Affiliations:** State Key Laboratory of Lithospheric and Environmental Coevolution, Institute of Geology and Geophysics, Chinese Academy of Sciences, Beijing 100029, China; State Key Laboratory of Lithospheric and Environmental Coevolution, Institute of Geology and Geophysics, Chinese Academy of Sciences, Beijing 100029, China; State Key Laboratory of Lithospheric and Environmental Coevolution, Institute of Geology and Geophysics, Chinese Academy of Sciences, Beijing 100029, China; State Key Laboratory of Lithospheric and Environmental Coevolution, Institute of Geology and Geophysics, Chinese Academy of Sciences, Beijing 100029, China; State Key Laboratory of Lithospheric and Environmental Coevolution, Institute of Geology and Geophysics, Chinese Academy of Sciences, Beijing 100029, China; Key Laboratory of Earth and Planetary Physics, Institute of Geology and Geophysics, Chinese Academy of Sciences, Beijing 100029, China; Key Laboratory of Earth and Planetary Physics, Institute of Geology and Geophysics, Chinese Academy of Sciences, Beijing 100029, China; State Key Laboratory of Lithospheric and Environmental Coevolution, Institute of Geology and Geophysics, Chinese Academy of Sciences, Beijing 100029, China; State Key Laboratory of Lithospheric and Environmental Coevolution, Institute of Geology and Geophysics, Chinese Academy of Sciences, Beijing 100029, China; State Key Laboratory of Lithospheric and Environmental Coevolution, Institute of Geology and Geophysics, Chinese Academy of Sciences, Beijing 100029, China; State Key Laboratory of Lithospheric and Environmental Coevolution, Institute of Geology and Geophysics, Chinese Academy of Sciences, Beijing 100029, China; State Key Laboratory of Lithospheric and Environmental Coevolution, Institute of Geology and Geophysics, Chinese Academy of Sciences, Beijing 100029, China; Key Laboratory of Earth and Planetary Physics, Institute of Geology and Geophysics, Chinese Academy of Sciences, Beijing 100029, China; State Key Laboratory of Lithospheric and Environmental Coevolution, Institute of Geology and Geophysics, Chinese Academy of Sciences, Beijing 100029, China; State Key Laboratory of Lithospheric and Environmental Coevolution, Institute of Geology and Geophysics, Chinese Academy of Sciences, Beijing 100029, China

**Keywords:** Chang'e-6, SPA basin, Apollo basin, norite, impact melt sheet, lunar evolution

## Abstract

As the largest and oldest well-preserved impact structure on the Moon, the South Pole–Aitken (SPA) basin on the lunar farside is critical for understanding early solar system dynamics and lunar history, but accurately determining its age remains challenging. Crater-counting chronology and Apollo sample studies propose various SPA-forming ages, which require validation by *in*  *situ* sampling of the SPA basin. Here, we present the petrology, geochemistry and chronology of norite clasts from the SPA basin that were returned by Chang'e-6. These norites have highly anorthite-rich, rare-earth element-poor plagioclase and magnesium-rich pyroxene, in contrast to Mg-suite norites that were returned from the lunar nearside. Abundant Fe–Ni metals with meteoritic Ni/Co ratios, depletion of volatile elements and variable grain sizes and cooling rates strongly indicate that the norites were crystallized from an impact melt sheet. Precise Pb–Pb ages of zirconium-bearing minerals in the norites yield two distinct impact events at 3.87 and 4.25 Ga. The former represents an impact-resetting event within the basin. The latter finding is most consistent with the age of the SPA impact, providing an initial 4.25-Ga anchor for the older end of the lunar crater chronology and refining the timeline for early lunar evolution.

## INTRODUCTION

The ∼2500-km-diameter South Pole–Aitken (SPA) basin is the largest and oldest well-preserved impact structure on the Moon [1–3]. It is also one of the few ‘massive impact structures’ or post-accretion impact structures of >1000 km in diameter to be found throughout the solar system. The SPA massive impact therefore serves as a crucial record of early solar system dynamics and it also likely profoundly shaped lunar history [4]. Determining the radioisotopic age of the SPA basin would provide a critical, initial anchor for lunar cratering chronology [5] and aid in dating other solar system bodies [6]. Crater-counting chronology, Apollo sample and meteorite studies have proposed ages for the SPA basin that range from 4.26 to >4.33 billion years ago (Ga) [3,7–12], but these estimates require validation by *in*  *situ* sampling from the SPA basin directly. Remote sensing has revealed that norites cover the SPA non-mare basin floor [13,14], which is interpreted as the Moon's lower crustal and/or upper mantle materials [13,15,16], or as the differentiation products of a large-impact melt sheet [14,17,18]. Sampling these non-mare materials can provide ground-truth evidence to test their origin and potentially define the timing of the SPA impact.

China's Chang'e-6 mission successfully returned ∼1935 grams of lunar farside soils. The landing site (named Statio Tianjiang) is situated at 41.625°S, 153.978°W in the southern mare region of the Apollo basin—the largest impact crater (∼492 km in diameter) within the northeast SPA basin (Fig. S1). In this study, we selected ∼1600 fragments (>200 μm) from two soil samples (CE6C0100JYFM002, 2 g; CE6C0200YJFM001, 3 g) allocated by the China National Space Administration. The fragments are composed of ∼32% basalt, ∼48% breccia, ∼15% glass and ∼5% non-mare lithic clasts. The non-mare lithic clasts are dominated by norite (>80%), with minor anorthosite. Given the potential origin of the norite clasts as impact ejecta from the SPA basin, as indicated by remote-sensing data [19–22], this study focuses on their characteristics.

## RESULTS AND DISCUSSION

### South Pole–Aitken norites (SPANs)

**Figure 1.**
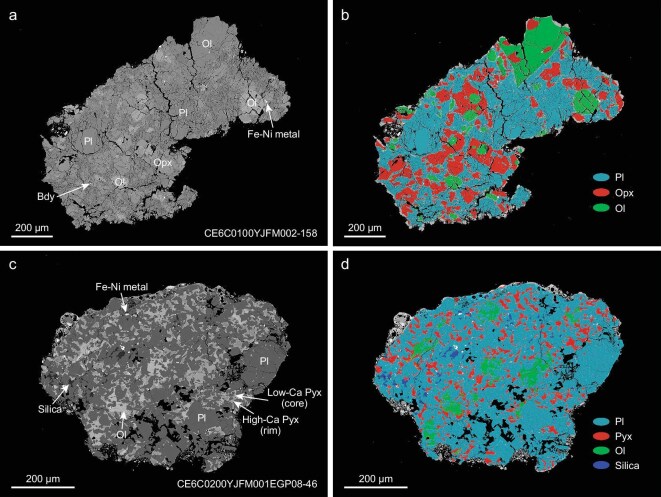
Two representative types of Chang'e-6 norite clasts. Backscattered electron images (a, c) and phase discrimination images based on X-ray mapping (b, d) are shown for comparison. (a, b) Coarse-grained norite clast has a poikilitic, intergranular texture with rounded olivine and subhedral orthopyroxene included in plagioclase. (c, d) Fine-grained norite clast showing interstitial, irregular olivine and pyroxene filling plagioclase. Ol, olivine; Opx, orthopyroxene; Pyx, pyroxene; Pl, plagioclase; Bdy, baddeleyite.

**Figure 2.**
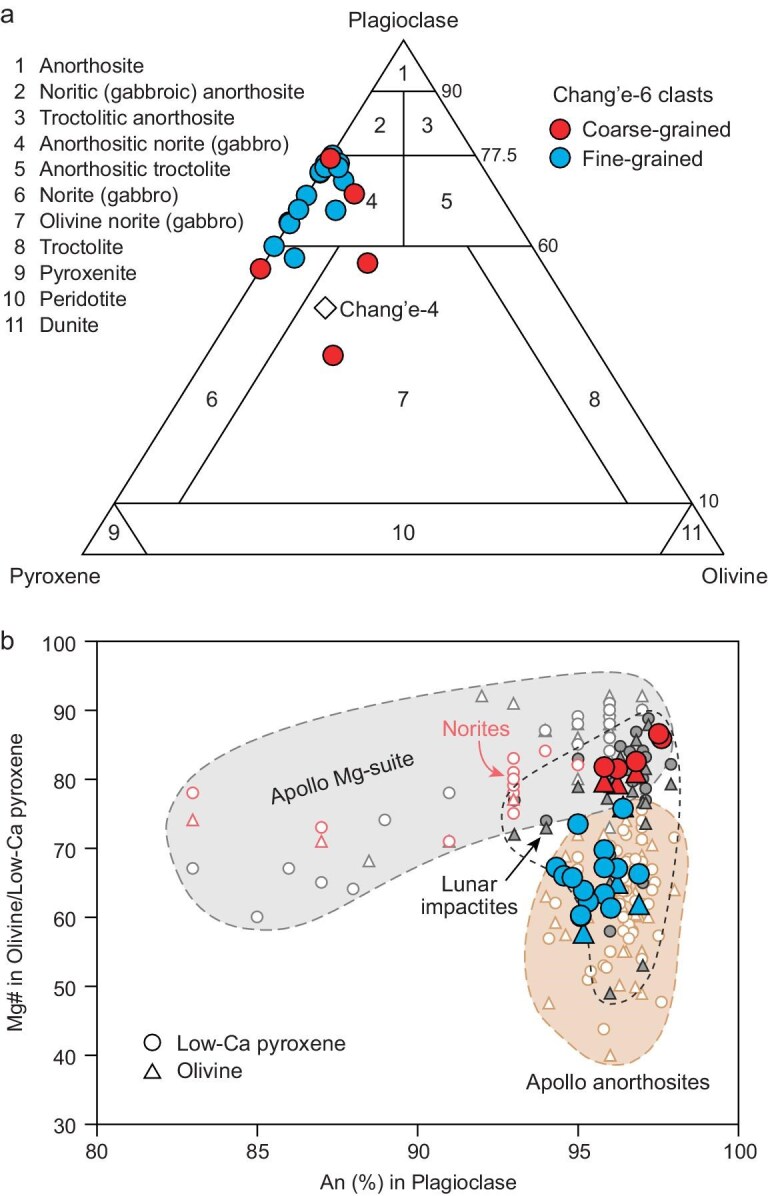
Lithological classification and mineral chemistry of Chang'e-6 norite clasts. (a) Model abundance of silicate minerals. Norite detected by Chang'e-4 [26] is also shown for comparison. (b) Composition of olivine, low-Ca pyroxene and plagioclase. Mineral compositions of Apollo Mg-suite rocks and ferroan anorthosites are from [27,28]. Lunar impactite data were compiled from [29–31].

Twenty representative norite clasts from the two Chang'e-6 samples studied were selected for petrological, geochemical and geochronological analyses (Table S1). The norite clasts exhibit various grain sizes and consist mainly of low-Ca pyroxene, plagioclase and olivine, with minor silica (cristobalite and tridymite), Fe–Ni metal, troilite, spinel and zirconium-bearing minerals baddeleyite, zirconolite, tranquillityite and zircon (Fig. 1, and Figs S2 and S3). Lithological classification diagrams indicate that these clasts are predominantly anorthositic norite, olivine norite and norite (hereafter referred to as norite clasts) in composition (Fig. 2a). Five coarse-grained (>100 μm) norite clasts have plutonic textures, with rounded olivine and subhedral orthopyroxene included in plagioclase (Fig. 1a and b) or maskelynite (a glassy material transformed from plagioclase during high-pressure impact). Both olivine and orthopyroxene are compositionally homogeneous (Fig. S4) with high magnesium number (Mg# = molar Mg/[Mg + Fe] × 100) values of 82–86 (Fig. 2b). The rest of the norite clasts have fine-grained (<30 μm), interstitial, anhedral pyroxene filling the plagioclase boundaries (Fig. 1c and d), which is distinct from plutonic textures in the lunar Mg-suite norites but similar to those in impact melt rocks [23–25]. The pyroxene grains in this type of norite clasts exhibit chemical zoning but are dominantly low-Ca pyroxene with a restricted Mg# of 60–76 (Fig. S4 and Table S2). Within both types of norites, euhedral-to-subhedral Fe–Ni metals occur as inclusions in olivine and plagioclase and as intergrowth phases with pyroxene (Fig. S5).

Although the Chang'e-6 norites show similar mineral constitution and bulk composition to the Apollo Mg-suite norites (Fig. S6 and Table S3), the former have distinct mineral compositions (Fig. S7). All plagioclase grains in the studied clasts are highly anorthite (An)-rich (An_94.3–97.6_), making the Chang'e-6 norites cross the fields of ferroan anorthosite (FAN) and Mg-suite troctolite but plot just outside of the range of Mg-suite norites, consistently with previously reported lunar impactites [29–31] (Fig. 2). Olivine and orthopyroxene in the coarse-grained norites have extremely low nickel (5.2–9.6 μg g^–1^) and cobalt (9.9–25.7 μg g^–1^) concentrations—even noticeably lower than those in Mg-suite rocks (Fig. S7). This feature may be attributed to the early crystallization of Fe–Ni metals [32] observed in the samples. In addition, the lower rare-earth element (REE) concentrations of orthopyroxene and plagioclase and lower P concentrations of olivine than those in Apollo and Luna Mg-suite norites [33] (Fig. S8 and Tables S4–S6) suggest either an insignificant KREEP (potassium [K], REE and phosphorus [P]) contribution to the genesis of the studied norites or considerable volatile loss during impacting. Therefore, the Chang'e-6 norites from SPA are distinct from intrusive Mg-suite norites that were returned from the lunar nearside.

### Evidence for impact melt

Several lines of evidence suggest that the studied SPANs were crystallized from impact melts. Firstly, impact melting would result in volatile element loss (such as Na, K, P and S), explaining the overall anorthite-rich plagioclase (An > 94), extremely low P in olivine (<193 μg g^–1^) and pyroxene (<83 μg g^–1^), and the scarce troilite in the norite clasts (Tables S1, S5 and S6). Secondly, the Fe–Ni metal with meteoritic Ni/Co ratios of 8.5–22.9 (Figs S5 and S7, and Table S7) suggests crystallization from melts that were contaminated by impactor composition [34,35] rather than from purely endogenic melts that contained scarce Fe-metal with non-meteoritic Ni/Co (mostly <5) [36]. Thirdly, both the presence of relict mineral clasts (Fig. S2g and k) and the interstitial pyroxene that fills the plagioclase within the fine-grained clasts are common textures in impact melt rocks [23,24]. In sum, the coarse-grained norite clasts have a meteoritic contamination signature, euhedral cumulative morphology for plagioclase and pyroxene, and intergranular texture that lacks mesostasis or interstitial glass—all features indicative of cumulate rocks from a large-impact melt sheet. This conclusion is supported by the negative correlations of pyroxene Mg# vs. plagioclase lanthanum and pyroxene ytterbium that are observed in the coarse-grained norite clasts (Fig. S8). The fine-grained norite clasts may represent crystalline melt rocks that derived from small impact events and/or those that were formed at shallow levels of a large-impact melt sheet. Therefore, the Chang'e-6 norite clasts are suitable candidates for dating impact events within the SPA basin.

### Ages of Chang'e-6 norites

**Figure 3.**
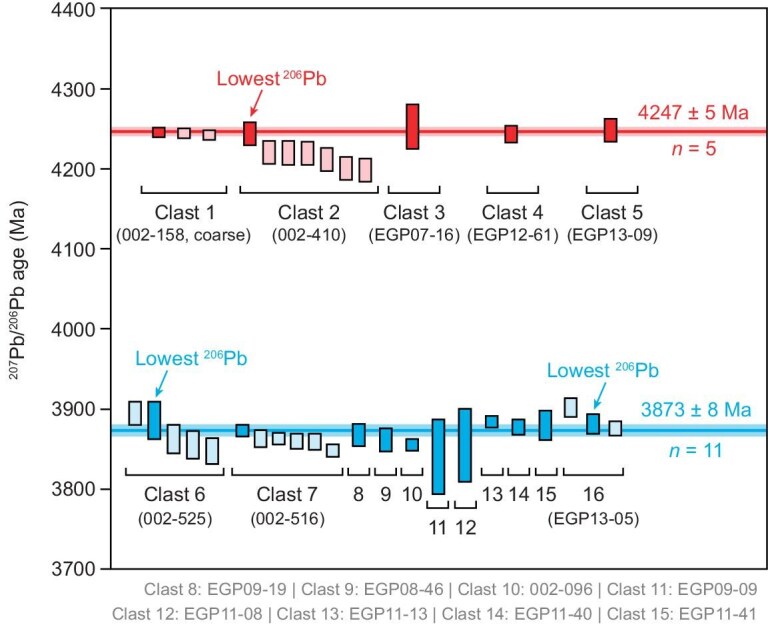
^207^Pb/^206^Pb ages of Zr-bearing minerals in Chang'e-6 impact melt norite clasts. The red and blue boxes represent the dates of the two old and young impact events, respectively. The dark-colored boxes are chosen as the best dates to calculate the average ages shown by the solid lines. The uncertainties of the average ages are plotted at 95% confidence. Box heights represent 2σ uncertainties.

To minimize potential disturbances from late impact events, we determined the formation ages of these impact melt rocks by using lead–lead (Pb–Pb) dating of Zr-bearing minerals with high uranium content, high closure temperature and near-zero initial Pb. We identified 33 baddeleyite, zirconolite, tranquillityite and zircon grains in 16 norite clasts (15 fine-grained and 1 coarse-grained; Table S8) that retained their primary crystalline texture, undisrupted by brecciation. All of these dated minerals occur as euhedral-to-subhedral grains that are intergrown with pyroxene and plagioclase (Fig. S3). To assess the reliability of each date, we performed multiple analyses on individual grains and/or multiple grains when present within a single clast. The ^204^Pb counts (average ∼ 0.01 counts s^–1^) and ^204^Pb/^206^Pb ratios (∼10^–5^ level) were sufficiently low, demonstrating a negligible effect on the common-Pb correction. Three dates on a 3 × 6 μm^2^ baddeleyite grain in coarse-grained clast 002–158 yield nearly identical ages (4247–4243 Ma), implying no detectable disturbance. Seven zirconolite grains, 1–2 μm in size, were found in clast 002–410. There is a ∼1% variation among the dating results. The grain with the lowest ^206^Pb counts, i.e. the lowest uranium content and the lowest radiogenic damage, yields the oldest age of 4245 ± 7 Ma (Fig. 3). Thus, we interpret this date as representing its formation age. The dating result of tranquillityite in clast EGP07-16 has less precision at 4254 ± 14 Ma due to its low ^206^Pb counts. Moreover, tranquillityite in clast EGP12-61 and zircon in clast EGP13-09 yield ages of 4244 ± 5 and 4250 ± 7 Ma, respectively. We adopt the five best dates to calculate an average of 4247 ± 5 Ma as the timing of an impact event (Fig. 3) because the other younger dates with elevated ^206^Pb counts might be slightly disturbed. The Zr-bearing minerals on the other 11 fine-grained clasts exhibit another distinct range of dates from 3903 to 3841 Ma, with an average of 3873 ± 8 Ma.

### SPA timing in solar system and lunar evolution

**Figure 4.**
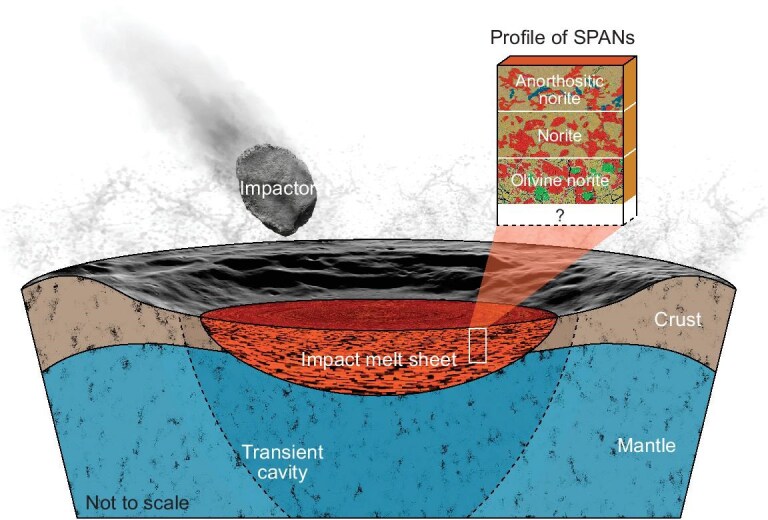
Formation of the SPA impact melt sheet and the lithologies identified by Chang'e-6. The 4.25-Ga SPA impact formed a large transient crater [15] with a melt sheet that formed through the melting of the lunar lower crust and the upper mantle [17]. Differentiation of the resulting melt sheet could have formed the SPAN layers that range from olivine norite to anorthositic norite, as observed in this study, with putative deeper materials likely being coarser-grained and having higher olivine abundances.

Our geochronological study of the Chang'e-6 SPANs reveals two distinct impact events (Fig. 3). The older event, dated at 4.25 Ga, is represented by five norite clasts with varying grain sizes and textures (Fig. 1, and Figs S2 and S3). This textural diversity likely reflects various cooling rates within different depths of a lunar impact melt sheet [25] that are similar to those observed in the largest terrestrial impact structures, such as Manicouagan [37], Sudbury [38] and Vredefort [39]. The presence of olivine, Mg-rich bulk composition (Mg# = 82.6), plutonic texture and chemically homogeneous pyroxene and olivine in the 4.25-Ga coarse-grained norite clast are collectively indicative of cumulates that were potentially formed relatively deep in the impact melt sheet. The other four fine-grained norites dated at 4.25 Ga show the presence of silica and more evolved compositions (lower Mg# of 61–66; Table S3), representing fractional crystallization products at shallower levels of the same impact melt sheet. We have discovered only one baddeleyite grain within five coarse-grained norites. The widespread occurrence of zirconium-bearing minerals in the fine-grained norites (Fig. S3) further demonstrates preferential enrichment of incompatible elements at shallow levels, consistently with prior theoretical predictions [17]. Therefore, our findings provide sample-based support for the formation of a large-impact melt sheet with differentiation layers that range from lower olivine norite to upper anorthositic norite (Fig. 4). The overall high Mg# values of the 4.25-Ga norites point to a highly magnesian primary composition for the impact melt sheet, suggesting a substantial contribution of mantle materials to its source. This inference indicates a massive impact that could have penetrated the crust–mantle boundary, melting the upper lunar mantle to form a large melt sheet, such as the SPA [14,17,18] and Orientale [40] impacts.

Our detailed geological survey reveals that the 4.25-Ga coarse-grained olivine norite was most likely ejected from SPA olivine norite regions, located at the southern periphery of the Apollo basin near the Chang'e-6 landing site (Fig. S1). This interpretation is supported by recent remote-sensing studies [19,20,22], the discovery of olivine norite within the Von Kármán crater by the Yutu-2 rover of the Chang'e-4 mission [26] and our own comparative lithological analysis (see Supplementary Texts). We also identified in Chang'e-6 soils Mg-suite and alkali-suite clasts that formed at 4.34–4.31 Ga (Fig. S9). These clasts, showing mineral compositions that are similar to crustal intrusive rocks but distinct from the SPANs and lunar impactites, most likely represent endogenic magma activity that was unrelated to the SPA impact (see Supplementary Texts). In addition, large-impact craters (>300 km in diameter) within or surrounding the SPA basin all exhibit crater size-distribution model ages of <4.1 Ga: the Apollo basin at 4.1–3.9 Ga [3,10], the Orientale basin at ∼3.8 Ga [41], the Schrödinger basin at ∼3.8 Ga [10] and the Korolev basin at ∼3.9 Ga [42]. As the oldest impact melt rocks found in Chang'e-6 samples, the five ancient norite clasts most likely represent the SPA melt-sheet ejecta that were excavated from different depths and then redistributed at the landing site by later impacts. Thus, the older age of 4.25 Ga acquired here can be taken as the timing of the SPA impact.

The younger group suggests an impact event at ∼3.87 Ga that reset preexisting impact melt rocks or igneous rocks and formed most of the fine-grained SPANs. This age appears broadly consistent with the sample-based chronology for the Imbrium basin on the nearside (3.92–3.85 Ga) [43,44] and crater size-distribution model ages for the aforementioned Apollo, Orientale, Schrödinger and Korolev basins near the landing site. Thus, to explicitly link this impact age to a specific basin is challenging. Nevertheless, the observed predominance of 3.87-Ga fine-grained norites (11 out of 16 clasts) favors their origin as proximal ejecta rather than distally reworked materials that were subjected to extended surface processing. All these younger fine-grained SPANs are anorthositic norites, petrologically aligning with the non-mare materials within the Apollo basin [21,45] (see Supplementary Texts and Fig. S1 for details). They show a range of bulk Mg# values (55–75; Table S3) that are broadly consistent with Moon Mineralogy Mapper (M^3^) data for the Apollo basin [19,46]. As the Chang'e-6 landing site is located in the south mare basalt area of the Apollo basin, most norite ejecta were likely sourced from inside this basin [19,20]. Therefore, we propose that the major age peak of 3.87 Ga possibly represents the timing of the Apollo impact.

This study offers evidence that the largest preserved impact on the Moon occurred ∼320 million years after the beginning of the solar system, providing a critical, initial, sample-based farside anchor for lunar cratering chronology [3]. The radiometric ages of returned samples are the cornerstone of the crater-counting chronology that has been widely used for lunar surface dating. However, the current crater-counting model is solely based on samples that have been returned from the lunar nearside. The Chang'e-6 SPANs identified in this study represent the first *in*  *situ* farside sample sourced from the oldest well-preserved basin on the Moon. Therefore, the precise formation age of the SPA basin as determined here could serve as a ‘golden spike’ for the older end of the lunar crater chronology function [47], helping to determine the surface ages of ancient regions and early lunar impact history [11]. Using the crater-counting chronology models that are calibrated by dated lunar nearside samples, the calculated model ages of the SPA basin range from ∼4.31 to 4.26 Ga [1,3,10,11]. These results are surprisingly consistent with the radioisotope age determined here, implying that early impact fluxes on either side of the Moon were largely comparable.

The temporal sequence of the SPA impact and cumulate mantle overturn following lunar magma ocean solidification remains uncertain [48]. Chronological constraints indicate that Apollo FAN and Mg-suite rocks have ages that are concentrated around ∼4.35 Ga [49], which have previously been interpreted as records of either the final stage of lunar magma ocean solidification [50], a Moon-wide thermal event such as mantle overturn [27,49], tidal heating [51] or the SPA impact [9,23]. Although the last hypothesis has been recently suggested on the basis of a pronounced peak of precise zircon U–Pb dates from Apollo samples, our new findings only identify one zircon grain among 33 Zr-bearing minerals analysed within the SPANs. Impact models also predict that a very limited fraction of SPA melt-sheet materials could have been redistributed to nearside Apollo landing sites [51,52]. Furthermore, our direct dating of the SPA basin itself demonstrates that the Moon's largest impact is ∼100 million years younger than the ∼4.35-Ga event, thereby ruling out the last hypothesis for its origin. Thus, the definitive age of 4.25 Ga for the SPA basin provides a firm basis for the construction of a more complete temporal sequence of early lunar evolution.

## Supplementary Material

nwaf103_Supplemental_Files
