# Supplementary material for: South Pole–Aitken massive impact 4.25 billion years ago revealed by Chang'e-6 samples
Source: Natl Sci Rev. 2025 Mar 20;12(6):nwaf103. doi: 10.1093/nsr/nwaf103 (PMC12086667; doi:10.1093/nsr/nwaf103)
Supplement: nwaf103_Supplemental_Files [file nwaf103_supplemental_files.zip › Supplementary_Texts_Methods_Figures.docx]

**Supplementary Materials for**

**South Pole–Aitken massive impact 4.25 billion years ago revealed by Chang’e-6 samples**

**This file includes:**

**1. Supplementary Texts**

- Comparative lithological analysis
- Mg-suite and alkali-suite endogenic magmatism

**2. Methods**

- Sample preparation
- SEM analysis and EDS mapping
- Electron probe microanalysis (EPMA)
- Laser Raman analysis
- *In-situ* mineral trace-element analysis
- Pb-Pb isotope analysis of Zr-bearing minerals

**3. Supplementary Figures S1-S10**

- Fig. S1. Geological context of the SPA basin and the Chang’e-6 landing site
- Fig. S2. Backscattered images showing microtextures of the Chang’e-6 norites
- Fig. S3. Back-scattered electron images of microtextures of Zr-bearing minerals
- Fig. S4. X-ray mapping images of two types of norite clasts
- Fig. S5. Back-scattered electron images of microtextures of Fe-Ni metals
- Fig. S6. Bulk major element compositions of the Chang’e-6 norite clasts
- Fig. S7. Mineral compositions of the Chang’e-6 norite clasts
- Fig. S8. Rare earth element (REE) distribution of plagioclase and pyroxene
- Fig. S9. Chang’e-6 magnesium-suite and alkali-suite clasts
- Fig. S10. Results of Raman analysis of minerals from the Chang’e-6 norite clasts

**Supplementary Texts**

**Comparative** **lithological analysis**

A lithologic map of the SPA basin conducted using high-resolution Kaguya Multiband Imager data is shown in Supplementary Fig. S1. The map shows that the Apollo basin is dominated by noritic anorthosite in the northeast and anorthositic norite in the southwest. The SPA basin exhibits a more diverse lithological suite, including olivine norite, norite, and gabbronorite, in addition to the two rock types found within the Apollo basin. The two distinct impact events at 4.25 Ga and 3.87 Ga established in this study suggest that the impact-melt noritic clasts originated from multiple craters/regions. The 3.87 Ga anorthositic norite clasts, dominant in the Chang’e-6 impact clast population, were likely ejected from adjacent areas within the southwest Apollo basin by younger impacts like Chaffee S crater (Supplementary Fig. S1c). This interpretation is supported by crater ejecta models showing that the Chaffee S crater ranks first in terms of the thickness of ejecta found at the Chang’e-6 landing site [1].

In contrast, the less abundant 4.25 Ga noritic clasts, particularly the coarse-grained olivine norites, exhibit a depletion of plagioclase and enrichment in olivine and pyroxene. These older clasts were more likely to originate from regions outside the Apollo basin, potentially from its southern periphery, where such olivine-rich norites are abundant (green areas in Supplementary Fig. S1b), within the central SPA compositional anomaly (SPACA). This interpretation is supported by recent geological surveys [1-3] suggesting that the Chang’e-6 landing site may have captured foreign ejecta from the SPACA from the impact of White crater (Supplementary Fig. S1c). The discovery of olivine norite within the Von Kármán crater by the Yutu-2 rover of the Chang’e-4 mission [4] further corroborates the presence of such a lithology within the SPACA. Moreover, lithological classification diagrams indicate a closer affinity of the 4.25 Ga coarse-grained olivine norites to the SPA basin than to the Apollo basin (Supplementary Fig. S1d, e). Therefore, our comparative lithological analysis links the 4.25 Ga impact to the SPA basin and the 3.87 Ga impact to the Apollo basin.

**Mg-suite and alkali-suite** **endogenic magmatism**

In addition to impact-related norite clasts, we identified one Mg-suite clast and one alkali-suite clast within the Chang’e-6 soils (Supplementary Fig. S9). The Mg-suite clast is primarily composed of plagioclase (An_88.9-91.6_), orthopyroxene (En_57.5-62.1_Wo_2.6-5.4_Fs_33.1-39.2_), and clinopyroxene (En_39.6-42.5_Wo_37.7-44.0_Fs_16.4-19.7_), with accessory spinel, troilite, and baddeleyite. This clast exhibits a plutonic texture and is classified as a Mg-suite gabbronorite according to the lithological classification scheme of Ref. [5]. The alkali-suite clast, also classified as a gabbronorite, comprises plagioclase (An_83.5-86.6_), clinopyroxene (En_29.1-34.5_Wo_16.3-37.0_Fs_33.4-54.6_), orthopyroxene (En_41.6-52.4_Wo_2.7-5.6_Fs_44.9-54.0_), K-feldspar, ilmenite, silica phase, troilite, and zircon. Plagioclase grains are moderately shocked, while clinopyroxene grains exhibit unshocked augite crystals with pigeonite exsolution lamellae. Both clasts have mineral compositions consistent with lunar crustal intrusive rocks [6] but are distinct from the SPANs and lunar impactites (Supplementary Fig. S9c), indicating an origin from endogenic magmatism unrelated to the SPA impact. ^207^Pb/^206^Pb dating of baddeleyite in the Mg-suite clast and zircon in the alkali-suite clast yields ages of 4,340 ± 4 Ma and 4,315 ± 6 Ma, respectively. The 4.34–4.31 Ga farside endogenic magmatism broadly matches the pronounced age peak of lunar zircons [7] and ages of Mg-suite rocks [8, 9] from Apollo nearside samples. This consistency supports a global melting event around 4.35 Ga, which has been recently interpreted as tidally-driven remelting predating the SPA massive impact [10].

**Methods**

**Sample preparation**

The Chang’e-6 samples studied are lithic clasts separated from two soil samples (CE6C0100JYFM002, 2 g; CE6C0200YJFM001, 3 g). Approximately 1,600 clasts were picked up with a sieve (aperture 200 μm) and then embedded in epoxy mounts and polished using a grinder. We identified 20 representative norite clasts for in-situ analyses. All analyses in this work were conducted in the Institute of Geology and Geophysics, Chinese Academy of Sciences in Beijing, China.

**Scanning electron microscopy analysis and energy dispersive spectrometry mapping**

High-resolution backscattered electron imaging and energy-dispersive X-ray spectroscopy analyses of the Chang’e-6 norite clasts were performed using a Zeiss Gemini 450 field-emission scanning electron microscopy. Petrographic observations was conducted at an accelerating voltage of 15 kV, a beam current of 2 nA, and a working distance of 8.5 mm. Quantitative mapping using energy-dispersive X-ray spectroscopy (Oxford instruments) was employed to determine the bulk major element composition of representative clasts, following the methodology detailed in Ref. [11]. The procedure includes X-ray map collection, raw count output, and data extract-transform-load. X-ray mapping parameters were aligned with petrographic observations, using a collection time of 500 μs and a dwell time of 8 ms per point, ensuring high spatial resolution through a step size <1 μm. Data processing involved a mineral classifier to label each point, followed by averaging the composition of all points within each mineral and recalculating mineral densities. The accuracy of the mineral classifier was validated using a database of lunar minerals, while the accuracy and precision of energy-dispersive spectroscopy mapping were verified through analyses of certified reference minerals (Micro-Analysis Consultants Ltd Standards). Void pixels were excluded before normalizing bulk compositions to 100%. The detection limit of bulk major elements is ~0.1 wt %, with precision and accuracy better than 1% and 2%, respectively.

**Electron probe microanalysis**

Major and minor element compositions of silicate minerals and Fe-Ni metals were determined using a CAMECA SXFiveFE electron microprobe. Wavelength-dispersive spectrometers were employed to analyze samples at an accelerating voltage of 15 keV, a beam current of 20 nA, and a focused beam. Peak counting times were set to 10 s. Daily calibration of the instrument was conducted using both natural and synthetic standards. For silicate mineral analysis, standards included: Na on albite; Mg on MgO; Al and K on K-feldspar; Si, Ca, and Mn on rhodonite; Fe on hematite; Cr on Cr_2_O_3_; Ni on Ni metal; and Ti on rutile. For metal analysis, reference standards comprised: P on apatite; Si on a silicon wafer; and Ti, Cr, Mn, Fe, Co, Ni, Cu, and Zn on pure metals. The peak overlap correction function of the CAMECA software was applied to mitigate interferences between Fe Kβ (*n* = 1) and Co Kα lines, as well as Co Kα (*n* = 3) and S Kα lines for metal analysis. Detection limits (3*σ* above background) ranged from 0.01–0.07 wt%. All data were processed using the phi-rho-Z matrix correction method in the CAMECA PeakSight software. Based on the analysis of internal laboratory standards, precision for major (>1.0 wt%) and minor (0.1–1.0 wt%) elements was better than 1.5% and 5.0%, respectively. All analysed data are provided in Supplementary Tables S9, 10.

**Laser Raman analysis**

Laser Raman analysis was conducted using a Witec alpha 300R confocal Raman microscope. The laser beam was focused on the sample surface by 50× Zeiss EC Epiplan (numerical aperture = 0.75) or 100× Zeiss EC Epiplan-Neofluar (numerical aperture = 0.9) objectives. Spectra were excited with 532 nm radiation from a semiconductor laser. Laser energy varied from 5.0–7.0 mW, depending on the mineral type, to ensure the detection of weak spectral features. The scattered signal was analyzed by a Peltier-cooled CCD detector after dispersion by 300 and 600 grooves/mm gratings. Raman shift regions of 110–1,500 cm^-1^ were examined. The instrument was calibrated using a silicon peak at 520.7 cm^-^¹. Spectral acquisition times ranged from 2–40 seconds, with a total of 25–50 accumulations collected for each measurement. Representative Raman spectral are provided in Supplementary Fig. S10.

***In-situ* mineral trace-element analysis**

The trace-element abundances of pyroxene, plagioclase, and olivine in norite clasts were performed using a high-repetition-rate Genesis GEO femtosecond laser coupled to an Agilent 8900 ICP-MS instrument [12]. Ablation was performed using 40 × 40 𝜇m^2^ rectangle ablating spots at 2 Hz with an energy of 4 J cm^-2^ for 45 s after measuring the gas blank for 25 s. A 25 s washout between analyses was done prior to each analysis. The carrier (He) and nebulizer gas (Ar) flows were optimized during spot ablation of NIST SRM 612 to obtain maximum signal intensities, while keeping ThO^+^/Th^+^ <3 ‰ and U/Th between 0.9 and 1.1. We use reference glasses NIST SRM 612 and 614 as the calibration reference material, while ^29^Si was chosen as the internal standard. MPI-DING GOR128-G and ARM-3 were used as the quality control reference material [13]. The Iolite4 program was used for data reduction [14]. All analysed data are provided in Supplementary Tables S4–6.

**Pb-Pb isotope analysis of Zr-bearing minerals**

The Pb-Pb dating on Zr-bearing minerals were conducted using a CAMECA IMS 1280HR SIMS, using operating and data processing procedures as those described by Ref. [15]. In order to minimize contamination, we cleaned the mounts of norite clasts using a fine (0.25 μm) diamond paste and ethanol prior to carbon coating. A ~3 μm beam size was used with an O^-^ primary beam at ~0.12 nA and an accelerated potential of -13 kV. Four Electron Multipliers were used to collect the necessary Pb isotopes simultaneously. A 10nA ^16^O^-^ primary beam was used for pre-sputtering over 120 seconds before each analysis. Ion images of ^96^Zr_2_^16^O_2_^+^ and Pb isotopes were generated within a 25 × 25 μm^2^ area to fix the position of Zr-bearing minerals. Measured compositions were corrected for common Pb using non-radiogenic ^204^Pb. Corrections are sufficiently small to be insensitive to the choice of common Pb composition, and an average of present-day crustal composition [16] is used for the common Pb assuming that the common Pb is largely surface contamination introduced during sample preparation. NIST SRM610 glass [17] was used to calibrate the relative yield of different Electron Multipliers and to evaluate the external reproducibility. Uncertainties on individual analyses in data tables are reported at a 1*σ* level; mean ages for pooled ^207^Pb/^206^Pb analyses are quoted with 95% confidence interval. Age calculations were carried out using the Isoplot/Ex program [18]. All analysed data are provided in Supplementary Tables S8, 11.


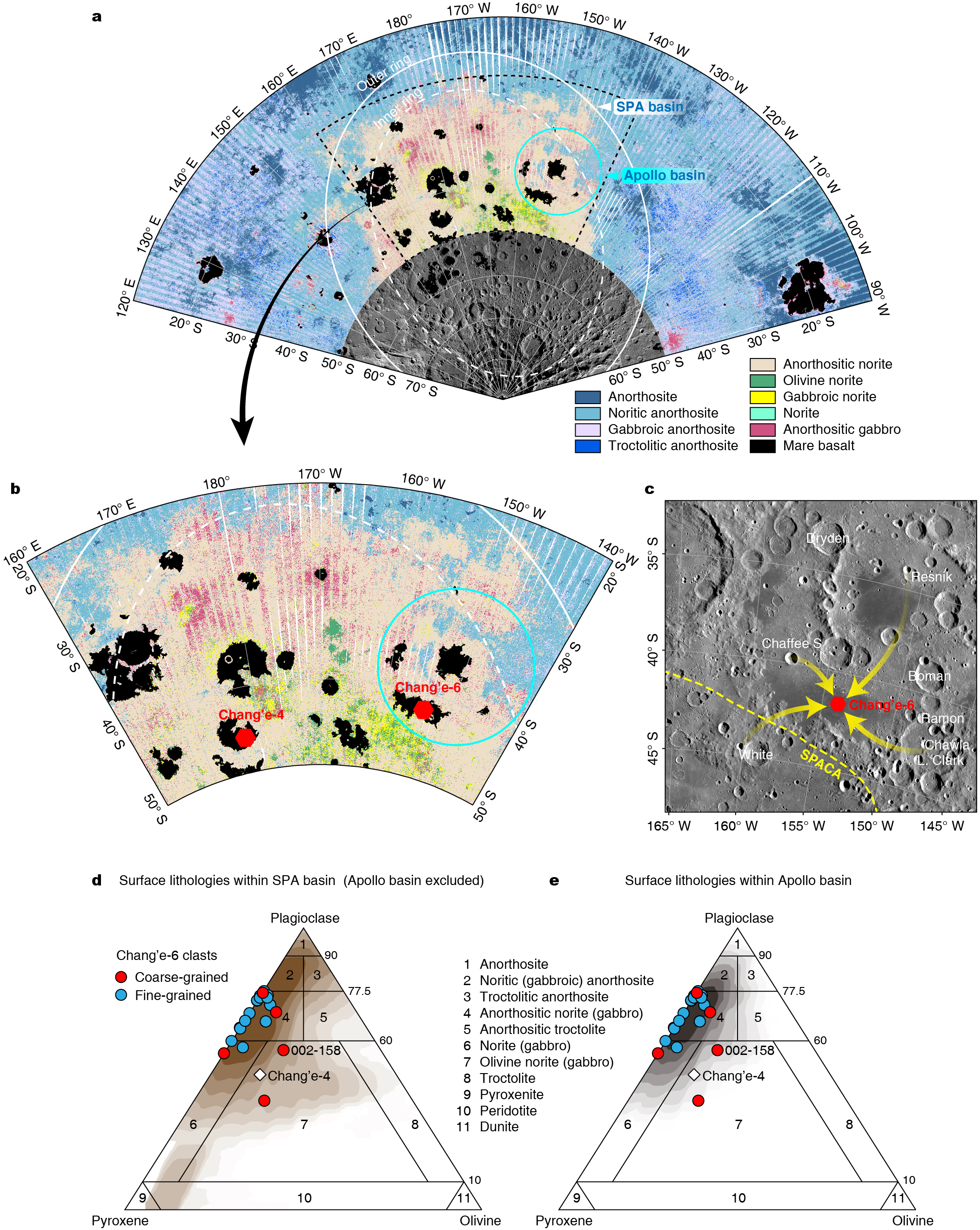


**Figure S1. Geological context of the SPA basin and the Chang’e-6 landing site. a, b**, Lithologic map of the SPA basin based on the lunar rock type classification scheme outlined in Ref. [5], with mare basalt area derived from Ref. [19] and mineral abundance data from Ref. [20]. The landing sites of Chang’e-4 and Chang’e-6 are also shown. The dashed and solid white circles represent the inner and outer rings of the SPA basin, respectively [21]. **c**, Image showing potential provinces of non-mare materials for the Chang’e-6 lunar soils [1-3, 22]. SPACA, SPA compositional anomaly. **d**, Model abundances of silicate minerals within the SPA non-mare regions (Apollo basin excluded) along with comparisons to the Chang’e-6 samples. **e**, Model abundance of silicate minerals within the Apollo basin non-mare region compared with the Chang’e-6 samples.


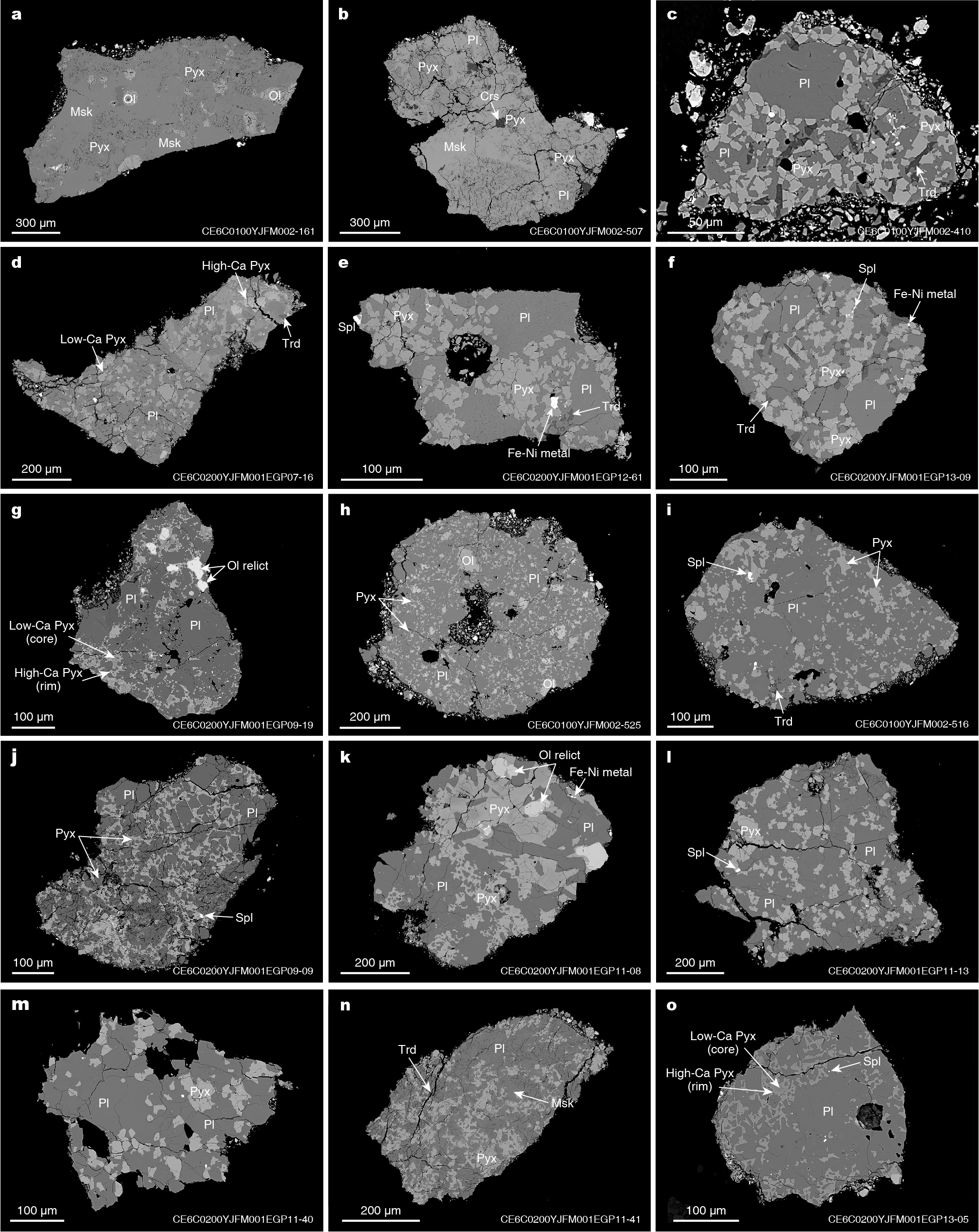


**Figure S2. Backscattered images showing microtextures of the Chang’e-6 norites. a**, A coarse-grained norite exhibiting a plutonic texture, with rounded olivine and subhedral orthopyroxene in maskelynite. **b**, Fractured orthopyroxene, plagioclase, and silica (cristobalite) in coarse-grained norite. Note that plagioclase was partially transferred to maskelynite. **c**, A 4.25 Ga fine-grained, poikilitic, intergranular norite has subhedral pyroxene and tridymite. **d**, A 4.25 Ga fine-grained norite dominated by plagioclase and low-Ca pyroxene with minor high-Ca pyroxene and tridymite. Plagioclase was partially transformed into maskelynite, forming veins in the upper right corner of the clast. **e**, A 4.25 Ga norite with plagioclase and pyroxene showing a cumulate texture. **f**, A 4.25 Ga norite exhibits a fine-grained, intergranular texture and contains subhedral pyroxene, plagioclase and tridymite. **g**, Interstitial, irregular-shaped pyroxene and relict olivine clast included in plagioclase within a fine-grained norite. The pyroxene has low-Ca cores and high-Ca rims, with Mg# values ranging from 59–73. The anhedral olivine surrounded by pyroxene, however, exhibits much lower Mg# values (49–55) than in pyroxene, pointing to relict mineral clasts. **h**, Anhedral olivine and interstitial pyroxene in a fine-grained norite. **i**, Interstitial pyroxene and silica (tridymite) filling the plagioclase boundary. Spinel occurs as inclusions in pyroxene. **j**, Poikilitic texture consisting of pyroxene surrounding plagioclase within a fine-grained norite. **k**, A 3.86 Ga norite showing a poikilitic texture, with subhedral to anhedral pyroxene, euhedral plagioclase, and an anhedral relict olivine clast. **l**, A fine-grained norite consisting of pyroxene, plagioclase, and spinel. **m**, A 3.88 Ga fine-grained norite showing a granoblastic texture. **n**, A 3.88 Ga fine-grained norite with interstitial pyroxene filling the plagioclase boundary. **o**, A 3.88 Ga norite exhibiting a poikilitic texture, with chemically zoned pyroxene in plagioclase. Ol, olivine; Pyx, pyroxene; Pl, plagioclase; Spl, spinel; Trd, tridymite; Msk, maskelynite; Crs, cristobalite.


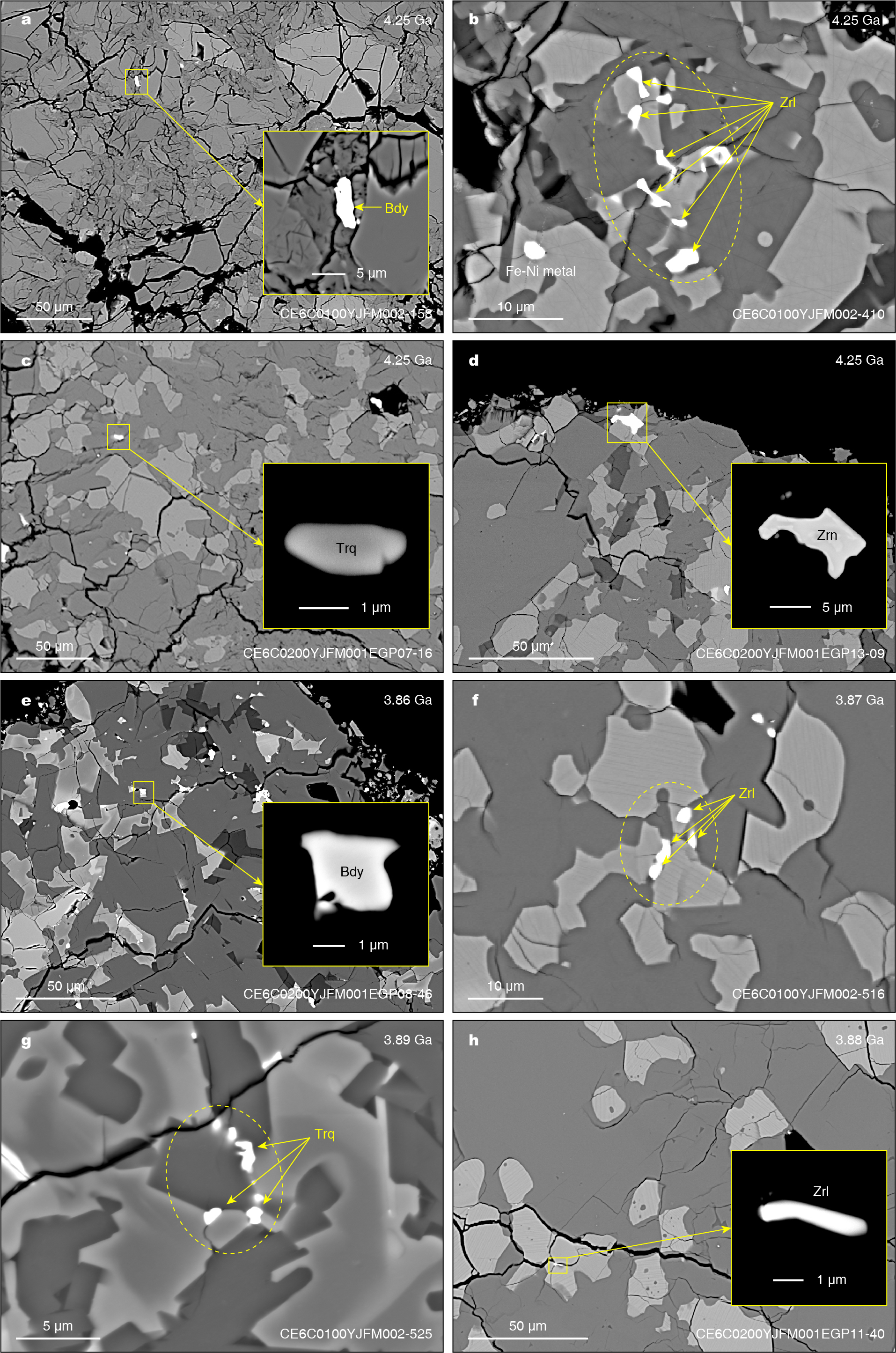


**Figure S3. Back-scattered electron images of microtextures of Zr-bearing minerals. a**, A 3 × 6 μm^2^ baddeleyite (Bdy) grain in a 4.25 Ga coarse-grained olivine norite. **b**, Intergrowth of zirconolite (Zrl) multi-grains and low-Ca pyroxene in a 4.25 Ga fine-grained norite. **c**, A tranquillityite (Trq) grain in a 4.25 Ga fine-grained norite. **d**, A zircon (Zrn) grain in a 4.25 Ga fine-grained norite. **e**, Euhedral baddeleyite included in plagioclase within a 3.86 Ga fine-grained norite. **f**, Intergrowth of zirconolite multi-grains and low-Ca pyroxene in a 3.87 Ga fine-grained norite. **g**, Intergrowth of tranquillityite multi-grains with low-Ca pyroxene in a 3.89 Ga fine-grained norite. **h**, Elongated zirconolite included in pyroxene within a 3.88 Ga fine-grained norite.


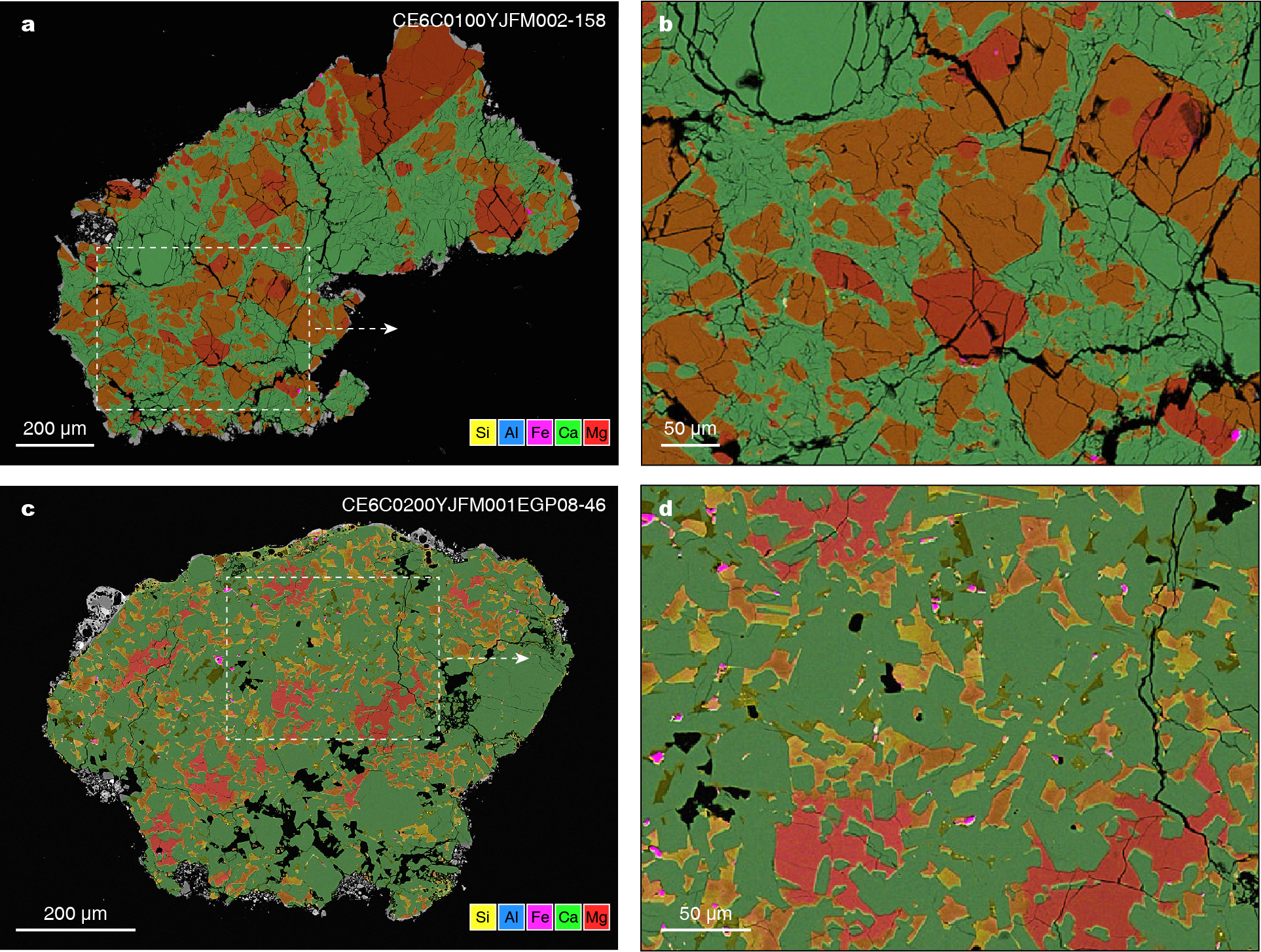


**Figure S4. X-ray mapping images of two types of norite clasts. a, b**, A 4.25 Ga coarse-grained olivine norite exhibits plutonic texture and compositionally homogeneous orthopyroxene. **c, d**, A 3.86 Ga fine-grained anorthositic norite shows poikilitic texture, with interstitial and chemically zoned pyroxene.


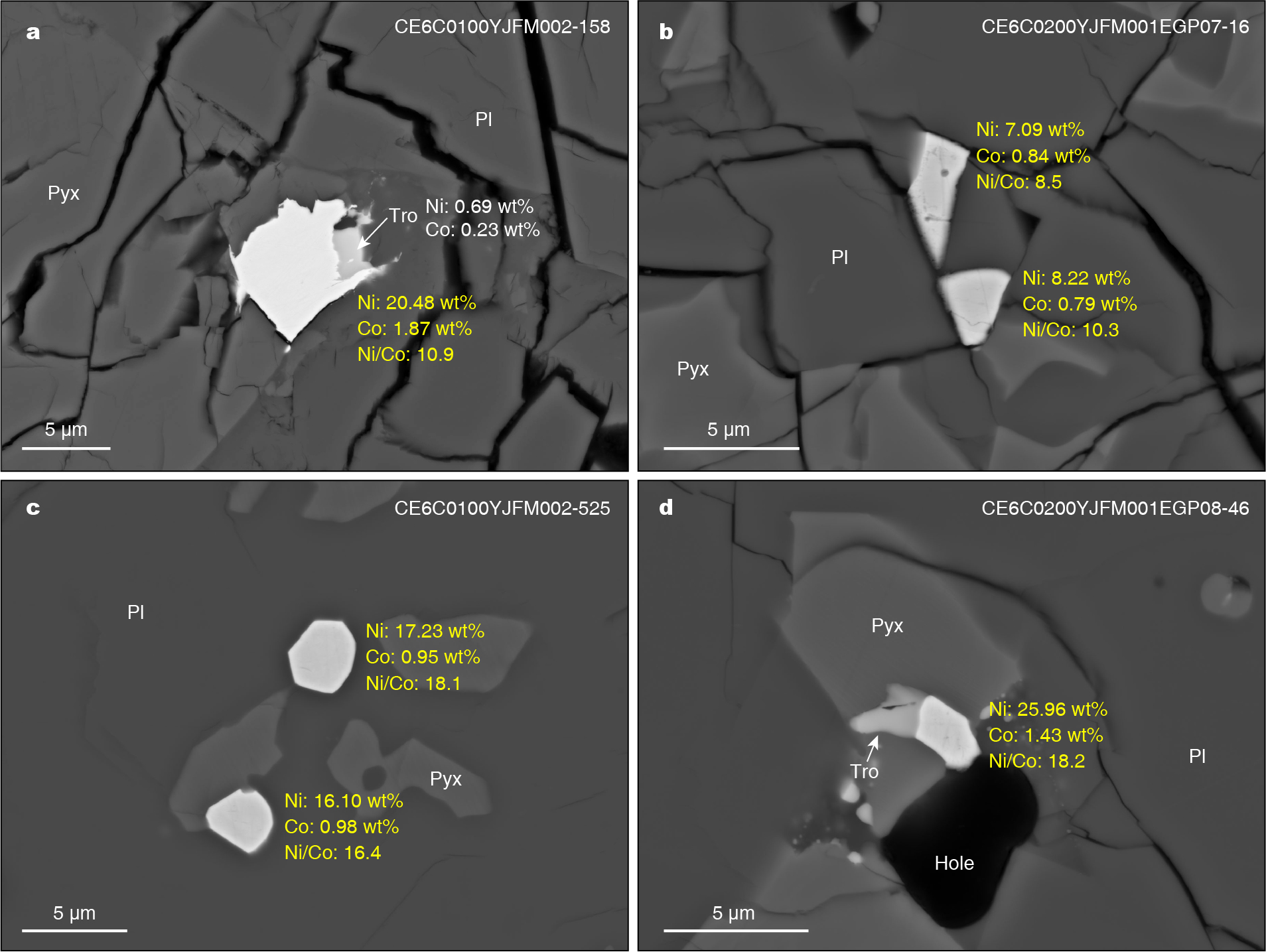


**Figure S5. Back-scattered electron images of microtextures of Fe-Ni metals.** **a**, Fe-Ni metal occurs as subhedral, intergranular phases associated with troilite and pyroxene within a coarse-grained norite that formed at ca. 4.25 Ga. **b**, Euhedral Fe-Ni metal is included within plagioclase in a fine-grained norite that formed at ca. 4.25 Ga. **c**, Euhedral Fe-Ni metals occur as inclusions in plagioclase and in contact with low-Ca pyroxene in a fine-grained norite (ca. 3.89 Ga). **d**, Intergrowth of Fe-Ni metal and troilite are included in pyroxene in a fine-grained norite (ca. 3.86 Ga).


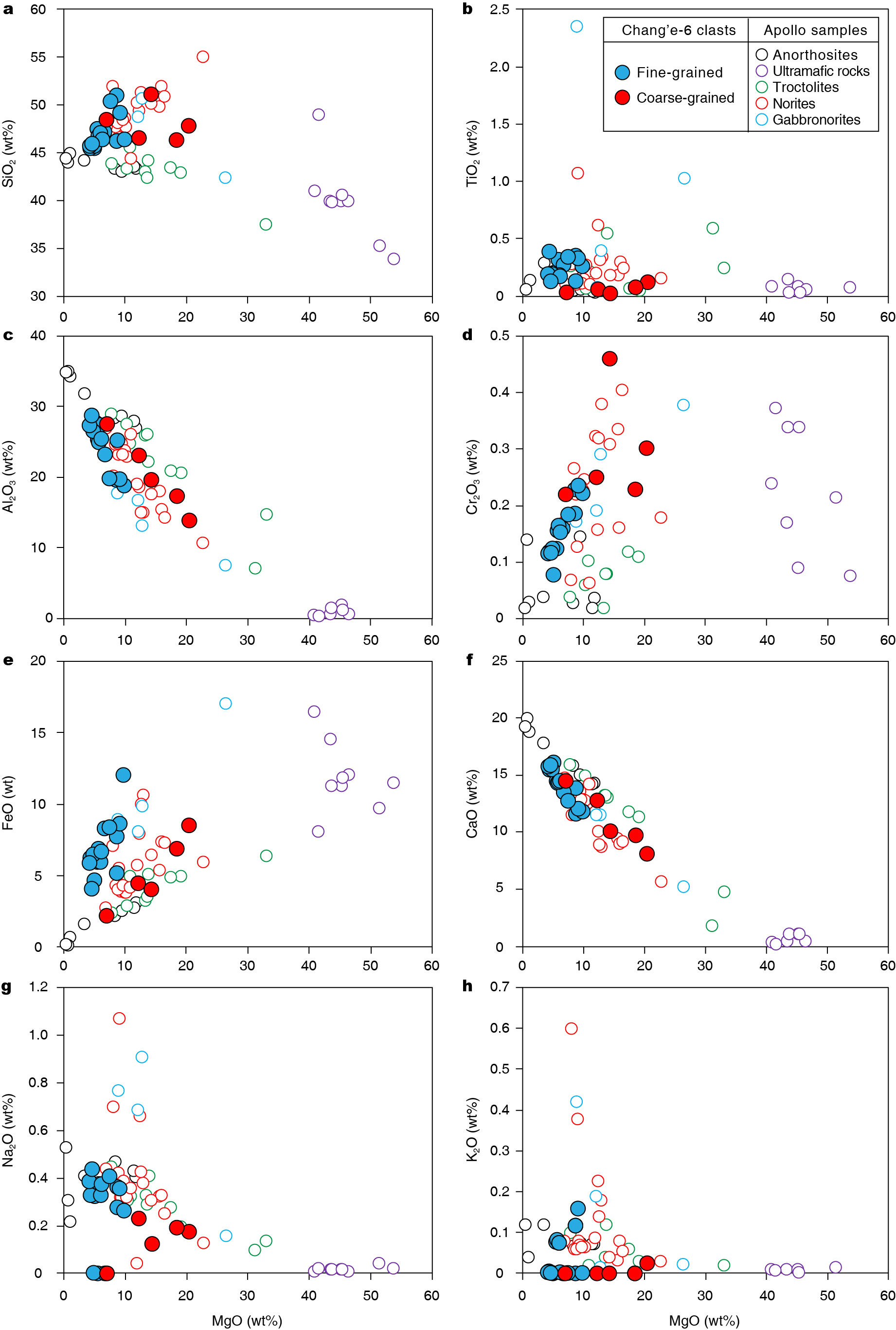


**Figure S6. Bulk major element compositions of the Chang’e-6 norite clasts.** For comparison, the compositions of Apollo Mg-suite rocks are also shown (see Source Data).


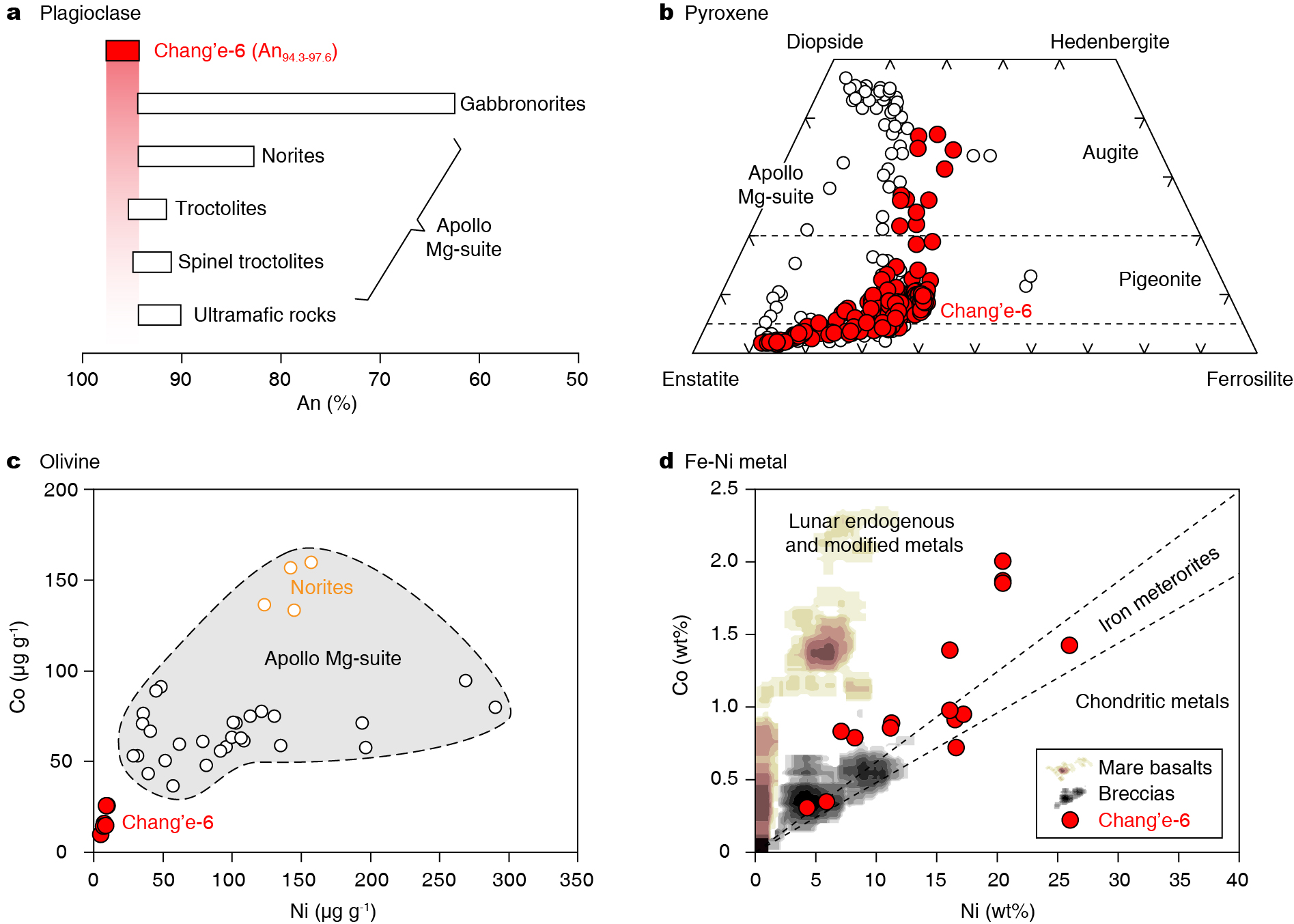


**Figure S7. Mineral compositions of the Chang’e-6 norite clasts. a**, Range in anorthite component (%) of plagioclase. **b**, Quadrilateral diagram of pyroxene. **c**, Olivine nickel vs. cobalt diagram. **d**, Nickel versus cobalt diagram for Fe-Ni metal grains. Data of Apollo mare basalts and breccias are shown based on kernel density estimation. Compositional ranges for chondritic metals and iron meteorites are from Ref. [23]. The Fe-Ni metals in the Chang’e-6 norites have similar compositions to those in Apollo breccias that were contaminated by exogenous impactors, but distinct from metals within endogenous mare basalts [23] and highlands rocks [24]. Note that several grains exhibit Ni and Co concentrations assembling within the ranges of iron meteorite and chondritic metals. Data for Apollo samples were obtained from plagioclase [6], pyroxene [6], olivine [25], and Fe-Ni metal [23].


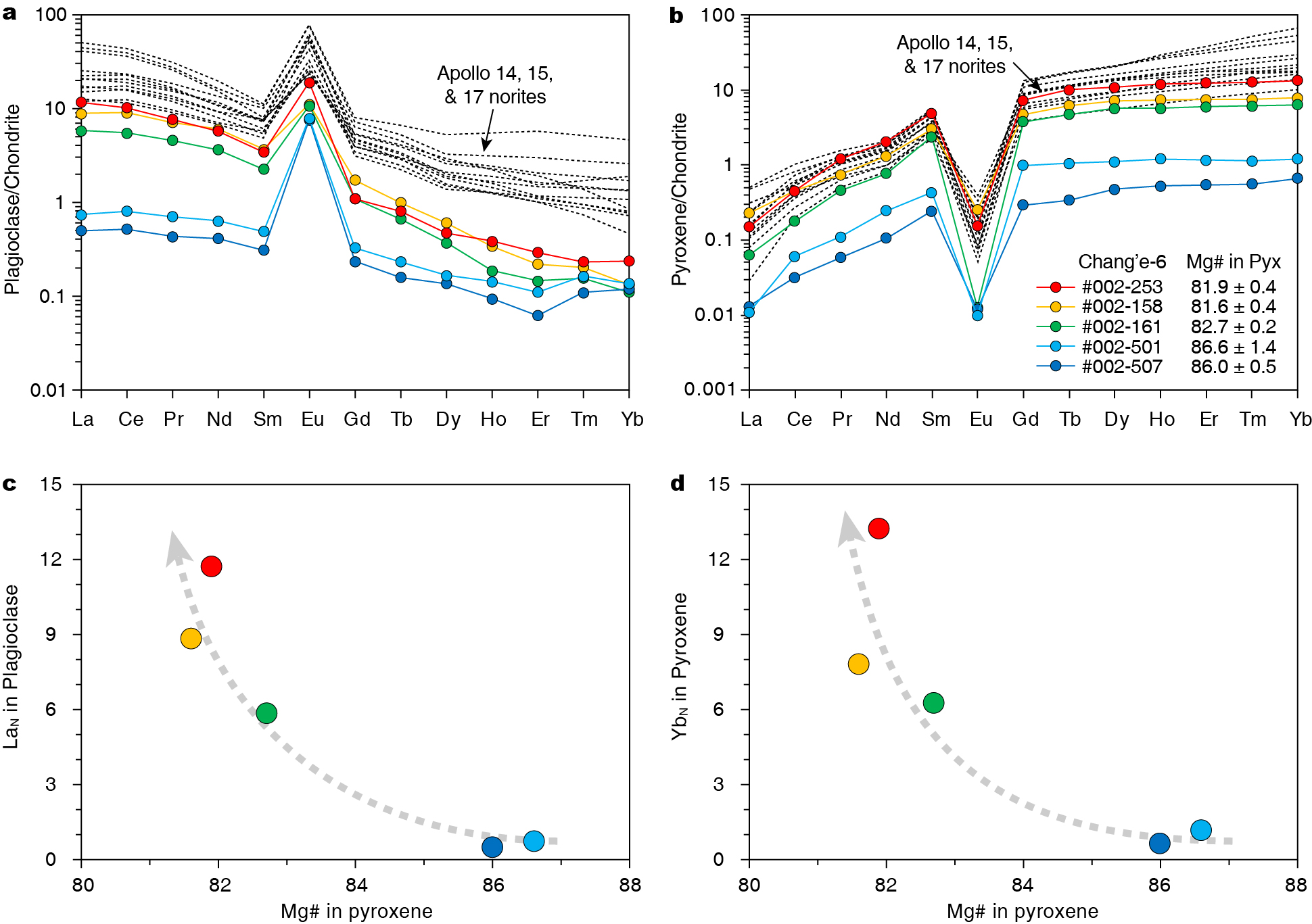


**Figure S8. Rare earth element (REE) distribution of plagioclase and pyroxene from the Chang’e-6 coarse norite clasts. a, b**, Chondrite normalized REE patterns. For comparison, the Apollo 14, 15, and 17 Mg-suite norite data are shown [26, 27]. **c**, Mg# in pyroxene versus chondrite normalized lanthanum in plagioclase. **d**, Mg# in pyroxene versus chondrite normalized ytterbium in pyroxene. The normalized data are from Ref. [28].


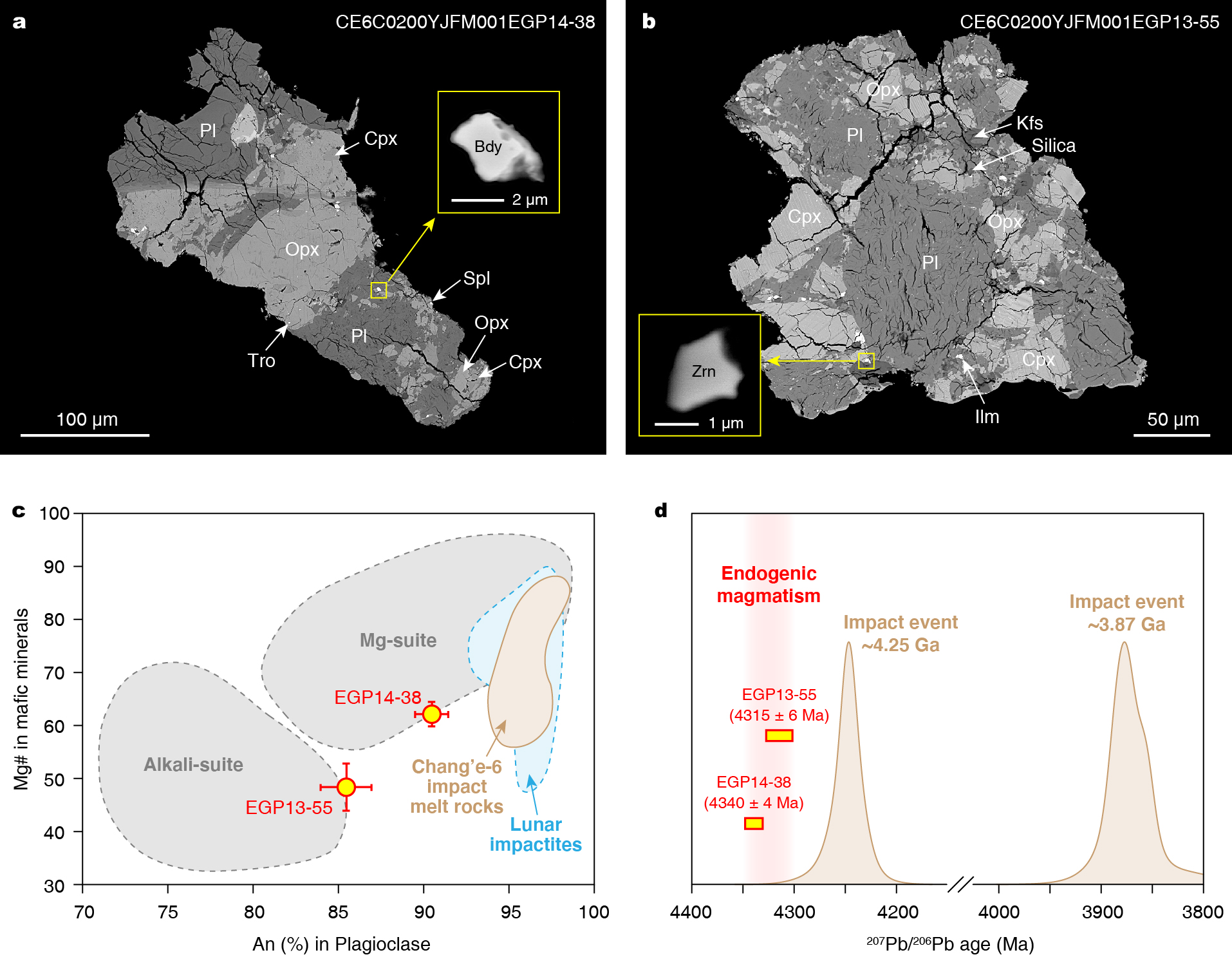


**Figure S9. Chang’e-6** **magnesium-suite and alkali-suite clasts.** Backscatter images showing microtextures of the Mg-suite (**a**) and alkali-suite (**b**) clasts. **a**, Mg-suite gabbronorite EGP14-38 has a plutonic texture and consists mainly of plagioclase, orthopyroxene, and clinopyroxene, with minor spinel, troilite, and baddeleyite. **b**, Alkali-suite gabbronorite EGP13-55 contains plagioclase, clinopyroxene, orthopyroxene, K-feldspar, ilmenite, silica phase, troilite, and zircon. Plagioclase grains are moderately shocked, while clinopyroxene grains exhibit unshocked augite crystals with pigeonite exsolution lamellaes. **c**, A plot of Mg# in mafic minerals versus anorthite content in plagioclase. Grey fields for Mg-suite and alkali-suite are from Ref. [6]. Error bars represent 1σ uncertainties. **d**, ^207^Pb/^206^Pb ages of Zr-bearing minerals in Chang’e-6 Mg-suite and alkali-suite clasts. Box widths represent 2σ uncertainties. The two impact events identified in Chang’e-6 norites are shown here for comparison. Pl, plagioclase; Opx, orthopyroxene; Cpx, clinopyroxene; Spl, spinel; Tro, troilite; Kfs, K-feldspar; Ilm, ilmenite; Bdy, baddeleyite; Zrn, zircon.


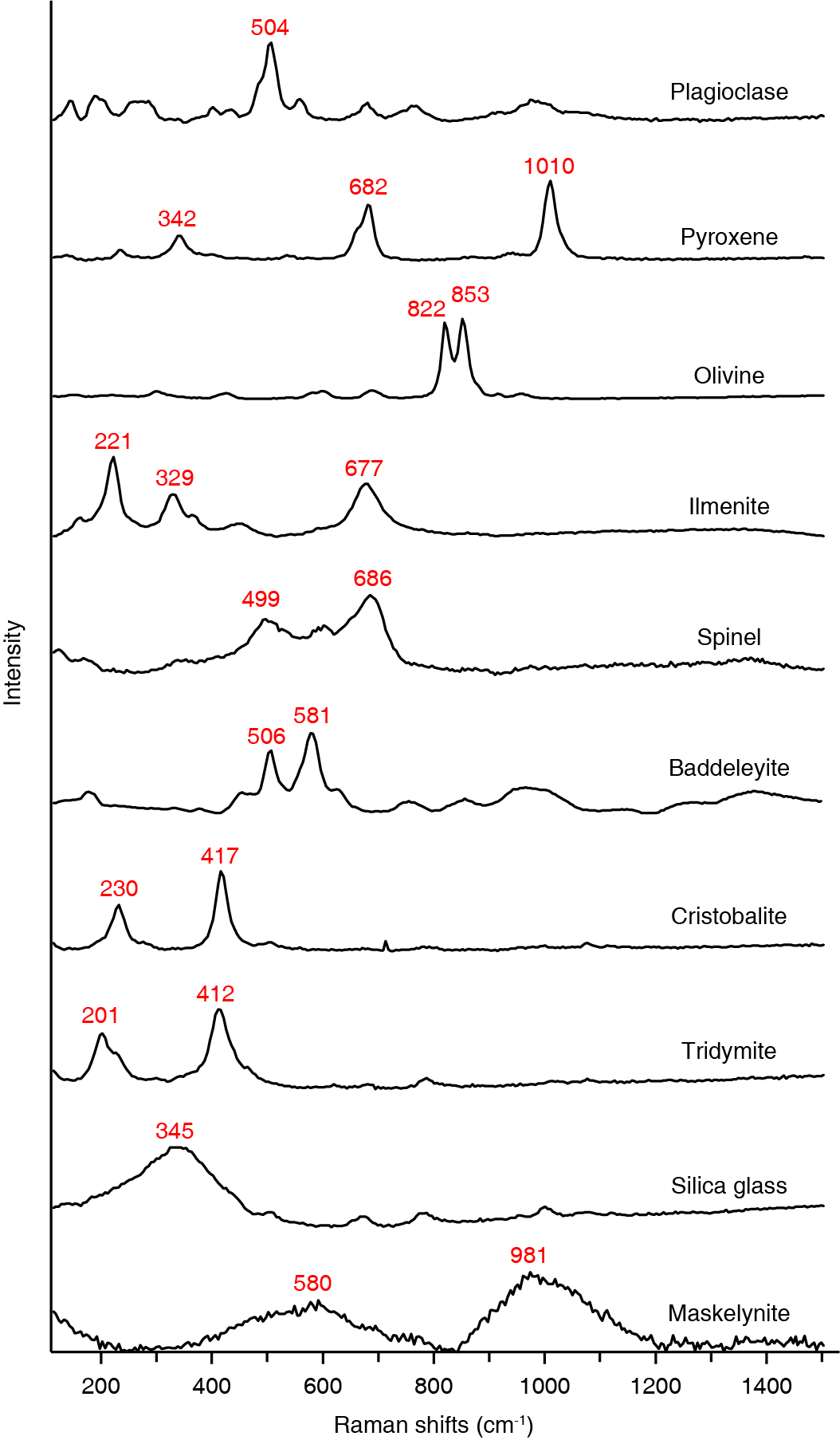


**Figure S10. Results of Raman analysis of minerals from the Chang’e-6 norite clasts.**

**REFERENCES**

1. Yue Z, Gou S, Sun S *et al.* Geological context of the Chang’e-6 landing area and implications for sample analysis. *The Innovation* 2024; **5**: 100663.

2. Jia Z, Chen J, Kong J *et al.* Geologic context of Chang’e-6 candidate landing regions and potential non-mare materials in the returned samples. *Icarus* 2024; **416**: 116107.

3. Xu L, Qiao L, Xie M *et al.* Chronology, local stratigraphy, and foreign ejecta materials at the Chang’e-6 landing site: Constraints on the provenance of samples returned from the Moon's farside. *Geophys Res Lett* 2024; **51**: e2024GL111311.

4. Lin H, He Z, Yang W *et al.* Olivine-norite rock detected by the lunar rover Yutu-2 likely crystallized from the SPA-impact melt pool. *Natl Sci Rev* 2020; **7**: 913–20.

5. Stöffler D, Knöll H, Marvin U *et al.* Recommended classification and nomenclature of lunar highland rocks-a committee report. *Lunar Highlands Crust* 1980: 51–70.

6. Shearer CK, Elardo SM, Petro NE *et al.* Origin of the lunar highlands Mg-suite: An integrated petrology, geochemistry, chronology, and remote sensing perspective. *Am Mineral* 2015; **100**: 294–325.

7. Barboni M, Szymanowski D, Schoene B *et al.* High-precision U–Pb zircon dating identifies a major magmatic event on the Moon at 4.338 Ga. *Sci Adv* 2024; **10**: eadn9871.

8. Borg LE and Carlson RW. The evolving chronology of moon formation. *Annu Rev Earth Planet Sci* 2023; **51**: 25–52.

9. Borg LE, Connelly JN, Cassata WS *et al.* Chronologic implications for slow cooling of troctolite 76535 and temporal relationships between the Mg-suite and the ferroan anorthosite suite. *Geochim Cosmochim Acta* 2017; **201**: 377–91.

10. Nimmo F, Kleine T and Morbidelli A. Tidally driven remelting around 4.35 billion years ago indicates the Moon is old. *Nature* 2024; **636**: 598–602.

11. Yuan J, Huang H, Chen Y *et al.* Automatic bulk composition analysis of lunar basalts: novel big-data algorithm for energy-dispersive X-ray spectroscopy. *ACS Earth Space Chem* 2023; **7**: 370–8.

12. Liu Y, Xue D-S, Li W *et al.* Trace elements determination by femtosecond LA-ICP-MS of 10 mg extraterrestrial geological samples prepared as lithium borate glasses. *J Anal At Spectrom* 2024; **39**: 2728–36.

13. Elatikpo SM, Li H and Sallau AK. Nature of fluid and genetic affiliation of the Bakoshi-Kundila Au deposit, Nigeria: Evidence from trace elements in hydrothermal quartz. *Ore Geol Rev* 2023; **160**: 105620.

14. Paton C, Hellstrom J, Paul B *et al.* Iolite: Freeware for the visualisation and processing of mass spectrometric data. *J Anal Atom Spectrom* 2011; **26**: 2508–18.

15. Li QL, Zhou Q, Liu Y *et al.* Two-billion-year-old volcanism on the Moon from Chang’e-5 basalts. *Nature* 2021; **600**: 54–8.

16. Stacey Jt and Kramers J. Approximation of terrestrial lead isotope evolution by a two-stage model. *Earth Planet Sci Lett* 1975; **26**: 207–21.

17. Baker J, Peate D, Waight T *et al.* Pb isotopic analysis of standards and samples using a ^207^Pb–^204^Pb double spike and thallium to correct for mass bias with a double-focusing MC-ICP-MS. *Chem Geol* 2004; **211**: 275–303.

18. Ludwig KR. Isoplot 3.6: a geochronological toolkit for Microsoft Excel (Berkeley Geochronology Center, 2008).

19. Nelson D, Koeber S, Daud K *et al.* Mapping lunar maria extents and lobate scarps using LROC image products. *Lunar Planet Sci* 2014; **45**: 2861.

20. Lemelin M, Lucey P, Gaddis L *et al.* Global map products from the Kaguya multiband imager at 512 ppd: Minerals, FeO, and OMAT. *Lunar Planet Sci* 2016; **47**: 2994.

21. Garrick-Bethell I and Zuber MT. Elliptical structure of the lunar South Pole–Aitken basin. *Icarus* 2009; **204**: 399–408.

22. Wang X, Head JW, Zhao W *et al.* Lunar Farside Samples Returned by Chang’E-6 Mission: Significance for Understanding the South Pole-Aitken Basin Stratigraphic History. *Astron J* 2024; **168**: 247.

23. Day JM. Metal grains in lunar rocks as indicators of igneous and impact processes. *Meteorit Planet Sci* 2020; **55**: 1793–807.

24. Ryder G, Norman MD and Score RA. The distinction of pristine from meteorite-contaminated highlands rocks using metal compositions. *Proc Lunar Planet Sci Conf* 1980; **11**: 471–9.

25. Shearer CK and Papike J. Early crustal building processes on the moon: Models for the petrogenesis of the magnesian suite. *Geochim Cosmochim Acta* 2005; **69**: 3445–61.

26. Papike J, Fowler G and Shearer C. Orthopyroxene as a recorder of lunar crust evolution: An ion microprobe investigation of Mg-suite norites. *Am Mineral* 1994; **79**: 796–800.

27. Papike J, Fowler G, Shearer C *et al.* Ion microprobe investigation of plagioclase and orthopyroxene from lunar Mg-suite norites: Implications for calculating parental melt REE concentrations and for assessing postcrystallization REE redistribution. *Geochim Cosmochim Acta* 1996; **60**: 3967–78.

28. McDonough WF and Sun S-S. The composition of the Earth. *Chem Geol* 1995; **120**: 223–53.
